# Mechanism of regulation of stem cell differentiation by matrix stiffness

**DOI:** 10.1186/s13287-015-0083-4

**Published:** 2015-05-27

**Authors:** Hongwei Lv, Lisha Li, Meiyu Sun, Yin Zhang, Li Chen, Yue Rong, Yulin Li

**Affiliations:** The Key Laboratory of Pathobiology, Ministry of Education, Jilin University, Changchun, 130021 China; College of Public Health, Jilin University, Changchun, 130021 China; College of Clinical Medicine, Jilin University, Changchun, 130021 China; Department of Molecular Pharmacology and Experimental Therapeutics, Mayo Clinic, Rochester, MN 55902 USA

## Abstract

Stem cell behaviors are regulated by multiple microenvironmental cues. As an external signal, mechanical stiffness of the extracellular matrix is capable of governing stem cell fate determination, but how this biophysical cue is translated into intracellular signaling remains elusive. Here, we elucidate mechanisms by which stem cells respond to microenvironmental stiffness through the dynamics of the cytoskeletal network, leading to changes in gene expression via biophysical transduction signaling pathways in two-dimensional culture. Furthermore, a putative rapid shift from original mechanosensing to *de novo* cell-derived matrix sensing in more physiologically relevant three-dimensional culture is pointed out. A comprehensive understanding of stem cell responses to this stimulus is essential for designing biomaterials that mimic the physiological environment and advancing stem cell-based clinical applications for tissue engineering.

## Introduction

Stem cells hold enormous potential for treating a broad spectrum of human diseases due to their multipotency [1-3]. Besides biochemical signals, stem cell maintenance and differentiation are regulated by biophysical aspects of the microenvironment, including mechanical loading, substrate material property and cell shape [4,5]. With the development of biomimetic substrates, new data continue to reveal more and more inspiring details of extracellular matrix (ECM) stiffness, which profoundly impacts on stem cell self-renewal and commitment [6-8]. ECM stiffness is usually represented by the elastic modulus or Young’s modulus. Generally, ECM stiffness, which matches the stiffness of native tissue, guides stem cell differentiation down corresponding tissue lineages. For instance, substrates approximating to the elastic moduli of brain (0.1 to 1 kPa), pancreas (1.2 kPa), cartilage (3 kPa), muscle (8 to 17 kPa) and bone tissue (25 to 40 kPa) direct stem cells, especially mesenchymal stem cells (MSCs), to commit to neurocytes, beta cells, chondrocytes, myoblasts and osteoblasts, respectively [6,9,10]. There is a growing interest in understanding how stem cells feel and respond to specific stiffness; how the mechanical cue is converted into intracellular signaling cascades; and how gene expression changes and stem cell fate are determined.

**Figure 1:**
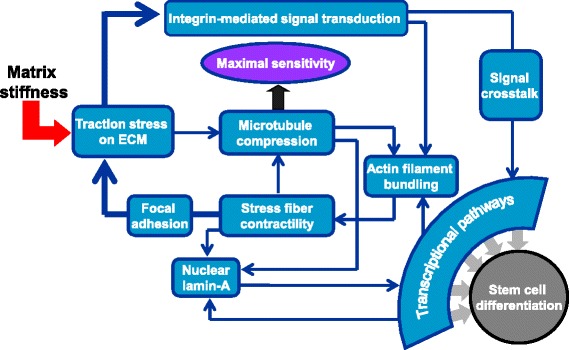
Mechanism of mechanotransduction of stem cells activated by matrix stiffness. The initial tension caused by stress fiber contraction is balanced by the microtubules resisting the resulting compression forces and the traction stress exerted on the extracellular matrix (ECM) across the focal adhesions, which directly cause the resultant force determined by matrix stiffness, contributing to microtubule compression. Then, the cell reads out the resultant forces from traction stress through the activation of integrin-mediated signal transduction pathways, which mediate actin filament polymerization and therefore change stress fiber contractility. Also, the initial tension from stress fiber contraction and the opposing compressive forces exerted by microtubules might also transmit into the nucleus and be resisted by lamin-A, which in turn promotes cell contractility by activating the transcriptional pathway that regulates actin filament bundling. Through cytoskeleton-based feedback loops, a cell changes its maximal mechanosensitivity close to the microtubule compression determined by matrix stiffness. Some transcriptional pathway modulates lamin-A expression, and feedback by lamin-A indirectly regulates transcriptional pathways, which crosstalk with integrin-mediated signaling and ultimately direct stem cell differentiation.

We summarize the mechanotransduction steps activated by matrix stiffness in stem cell differentiation (Figure 1). Based on recent experiments in two-dimensional models, the mechanism of mechanotransduction of stem cells is probably associated with the integrin-cytoskeletal-based feedback loop between mechanical signals and biochemical signals in the background of the signaling network to determine their fates. Different mechanisms of matrix stiffness mechanotransduction may exist in three-dimensional environments, which are more physiologically relevant.

## Integrin as the starting point for mechanosensing

The mechanical link between the ECM and the cytoskeleton are large protein assembly complexes known as focal adhesions, which involve integrins as major adhesion receptors [11]. Adapter proteins, including talin and vinculin, connect the cytoskeleton with integrins. Integrins are heterodimers comprising α and β subunits, each with multiple types. The binding affinity and specificity of these subunits for various ECM proteins are different, and they also play different roles in regulating the response of stem cells to mechanical properties of the microenvironment. For instance, β3 integrin mediates MSC myogenic differentiation induced by substrates with medium stiffness [12], while α2 integrin modulates osteogenesis of MSCs on stiff matrix [13]. As a vital cell surface transmembrane receptor, integrin mediates focal adhesion assembly, cytoskeletal organization, and a cascade of downstream signal transduction events through its activation during mechanotransduction [14]. Importantly, integrin transmits signals bidirectionally; that is, integrin not only transmits outside-in signals from the extracellular environment, but also passes intracellular stimuli to the outside of the cells [15]. The intracellular stimuli induce talin and kindlin to bind to the cytoplasmic domains of integrin β subunits, which activates the binding of integrins and ligands [16]. A growing number of studies suggest that mechanical stiffness modulates the self-renewal and differentiation of stem cells through integrin-mediated transduction pathways [13,17].

A host of intracellular signaling proteins are tightly associated with focal adhesions, such as focal adhesion kinase (FAK), a key component of mechanosensing. Under mechanical stimulation, the conformation of these signaling molecules may change, exposing phosphorylation sites and leading to activation of kinase cascades, transport of intracellular signaling molecules and changes in gene expression [18,19]. Among these signaling molecules, integrins are essential for mechanical signal perception, and are orientated at the start of the process of mechanotransduction induced by matrix stiffness [20].

## Integrin downstream signaling pathways

Integrin signaling interplays directly or indirectly with other pathways during mechanotransduction, including those involving RhoA, bone morphogenetic protein (BMP)/Smad and FAK. β3 integrin activity and RhoA signaling mediate the generation of cytoskeleton tension [12]. MSCs on micropatterned ECM with medium stiffness (10.2 kPa) preferentially recruit β3 integrin to develop larger and elongated focal adhesion complexes, which subsequently activate RhoA signaling [12]. It is known that RhoA activity is regulated by a wealth of GTP exchange factors or GTPase activating proteins (GAPs), including p190RhoGAP. The activation of RhoA leads to the stimulation of the contraction indicator myosin light chain kinase (MLCK), which cooperates with acto-myosin and actin filaments to promote stress fiber assembly to generate the appropriate cytoskeleton tension [21]. Additionally, β1 integrin internalization inhibits the BMP/Smad signaling pathway [17]. Soft substrates lead to a substantial increase in the active form of β1 integrin and a reduction in the cell surface distribution of β1 integrin in MSCs by enhancing the detachment of integrin-ECM protein complexes [17]. In turn, the detachment drives activated integrin internalization through caveolae/raft-dependent endocytosis. The enhanced uptake of β1 integrin subsequently influences membrane localization of BMP receptor (BMPR) by promoting BMPR endocytosis. This endocytosis prevents BMPR from binding to ligands and thus inhibits the phosphorylation of downstream Smad 1/5/8 [17]. Furthermore, the state switches in α5β1 integrin link the cytoskeleton to FAK pathways [20]. FAK is a fundamental signaling molecule in the integrin-mediated signal transduction pathway [22]. During the mechanosensing process, myosin II-modulated cytoskeletal tension triggers the α5β1 integrin to switch from a relaxed state to a tensioned state. The switch in integrin state directly monitors the α5β1-fibronectin bond strength via engaging the synergy site in fibronectin, and links the cytoskeleton to FAK pathways [20]. FAK signaling in turn promotes activation of integrin, leading to enhanced cell adhesion on ECM [23].

## Cytoskeletal-based feedback loops to reach maximal mechanosensitivity

Various studies have demonstrated that substrate stiffness-driven lineage specification of stem cells changes when actomyosin contractility is blocked or increased by pharmacological cytoskeletal inhibitors in two-dimensional culture [6,24-26] (Table 1). In other words, actomyosin contractility is critical for cells to perceive the stiffness of the microenvironment. Actomyosin contractility is modulated by actomyosin or myosin through sliding actin filaments in a polar fashion [27], and actomyosin filaments, known as stress fibers, mediate signal transduction from the ECM through integrins [28]. The response of the contraction rate or the contractile traction force results specifically from substrate stiffness and not cell tension or height. In an atomic force microscopy experiment [29], the cellular contraction rate, which represents the change in contractile traction force per unit time (dF/dt), rapidly increased while the corresponding contraction velocity, which is the change in cell height per unit time (dx/dt), decreased in response to increases in matrix stiffness. Focal adhesions, which represent dynamic actin-integrin linkage, provide essential ways of mechanical transmission of stiffness sensing through the contractility of stress fibers at the early stage of differentiation. Vinculin, a component of focal adhesion complexes, displays a more punctate structure on stiff substrates, but blocking of integrin-ECM binding by arginine-glycine-aspartate-serine (RGDS) leads to more diffuse vinculins and inhibits MSC osteogenesis on stiff matrix [26]. Stress fiber-focal adhesion complexes generate forces against the ECM, causing morphological change, migration and differentiation of the cell [30]. Furthermore, vimentin intermediate filaments, which are known to directly interact with actin, integrins, and their associated focal adhesions, may also act as mechanosensing elements as they become more punctate with increasing stiffness; and as vinculins, this punctate structure disappears when integrin-ECM binding is blocked [26].

**Table 1:** **Effects of pharmacological cytoskeletal inhibitors on stem cell differentiation induced by matrix stiffness**

**Chemical**	**Binding target**	**Mechanism**	**Stem cell types**	**Effects on differentiation**
Blebbistatin	Myosin ATPase	Inhibits actomyosin contractility by blocking non-muscle myosin II ATPase activity	MSCs	Two-dimensional: blocks neurogenesis at 0.1 to 1 kPa, myogenesis at 8 to 17 kPa, osteogenesis at 25 to 40 kPa [6]; suppresses chondrogenesis at 1 kPa [25]; inhibits osteogenesis at 47.5 kPa [24]
			Mammary progenitor cells	Two-dimensional: abrogates myoepithelial cell differentiation at 4 kPa [60]
			MSCs	Three-dimensional: no obvious effect on hypertrophic differentiation at approximately 53.6 kPa [48]; no obvious effect on osteogenesis at 0.2 to 59 kPa [46]
ML-7	Myosin light chain kinase	Inhibits actomyosin contractility by blocking myosin light chain phosphorylation	MSCs	Two-dimensional: blocks neurogenesis at 0.1 to 1 kPa, myogenesis at 8 to 17 kPa, osteogenesis at 25 to 40 kPa [6]
			MSCs	Three-dimensional: no obvious effect on osteogenesis at 0.2 to 59 kPa [46]
Y27632	ROCK	Inhibits actomyosin contractility by blocking the RhoA-ROCK pathway	Mammary progenitor cells	Two-dimensional: abrogates myoepithelial cell differentiation at 4 kPa [60]
			MSCs	Three-dimensional: no obvious effect on hypertrophic differentiation at ~53.6 kPa [48]; no obvious effect on osteogenesis at 0.2 ~ 59 kPa [46]
Calyculin A	Myosin light chain phosphatase	Increases actomyosin contractility by inhibiting the myosin light chain phosphatase	Mammary progenitor cells	Two-dimensional: increases luminal epithelial cell differentiation at 0.1 kPa [60]
Cytochalasin-D	Actin filament	Inhibits actin polymerization	ASCs	Two-dimensional: reduces the cellular area and aspect ratio of cells at 20 and 40 kPa and increases adipogenesis at 2 to 40 kPa, especially at 2 kPa [61]
Latrunculin A	Monomeric G-actin	Inhibits actin polymerization	MSCs	Three-dimensional: increases osteogenesis at 0.2 to 59 kPa [46]
Colchicine	Tubulin	Inhibits microtubule formation	MSCs	Three-dimensional: no obvious effect on osteogenesis at 0.2 to 59 kPa [46]

ASC, adipose-derived stem cell; MSC, mesenchymal stem cell; ROCK, Rho associated kinase.

**Figure 2:**
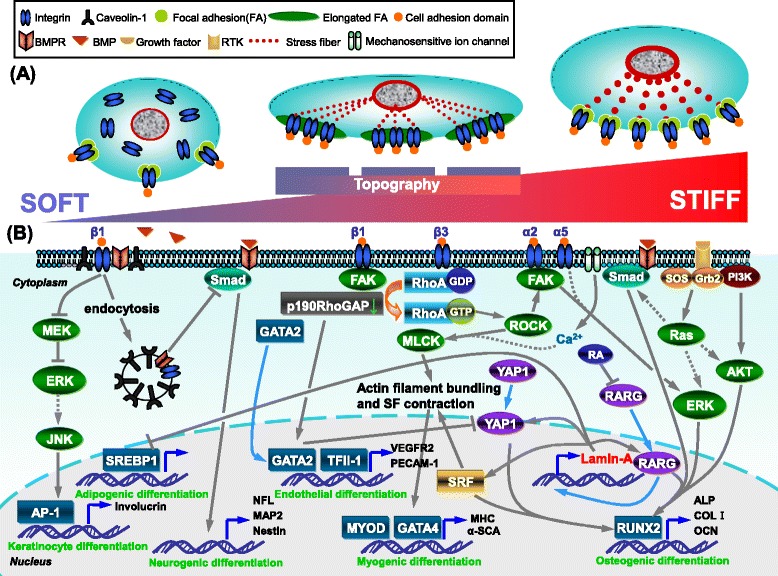
Stem cell response to matrix stiffness by integin, cytoskeleton and signal transduction crosstalk. **(A)** Integrin binding and cytoskeletal organization of stem cells seeded on substrates with varying stiffness. Left: on soft matrix stem cells present small spreading area, poorly defined actin cytoskeleton, low levels of lamin-A and detachment of focal adhesion complexes, associated with the uptake of integrins. Middle: on topographical substrates with medium stiffness, cells develop enlongated focal adhesions, intermediate levels of lamin-A, and well-aligned stress fibers with a spindle-shaped morphology. Right: stem cells cultured on rigid matrices display a large spreading size, prominent stress fibers and enhanced focal adhesion assembly, as well as high levels of lamin-A. BMP, bone morphogenetic protein; BMPR, bone morphogenetic protein receptor; RTK, receptor tyrosine kinase. **(B)** Crosstalk between signal transduction pathways induced by matrix stiffness to modulate stem cell lineage specification. Left: blocking of BMP/Smad signaling by enhanced uptake of β1 integrin through caveolae/raft-dependent endocytosis on soft matrix drives mesenchymal stem cell (MSC) neurogenic differentiation; lamin-A inhibits adipogenic differentiation by suppressing the , sterol regulatory element-binding transcription factor 1 (SREBP1) transcriptional pathway; blocking of integrin-mediated extracellular signal-regulated kinase (ERK)/mitogen-activated protein kinase (MAPK) signaling, which may activate AP-1 by stimulation of Jun N-terminal kinase (JNK), leads to keratinocyte differentiation of epidermal stem cells. Middle: on topographical substrates with medium stiffness, MSCs employ β3 integrin-RhoA-Rho associated kinase (ROCK)-myosin light chain kinase (MLCK) pathways to promote actin filament bundling and stress fiber contraction to create appropriate cytoskeletal tension, which further influences myogenic gene expression; medium stiffness acts through β1 integrin, causing a reduction of p190RhoGAP, which results in nuclear localization of GATA2 and TFII-1 in a RhoA-independent manner, ultimately leading to endothelial differentiation of cardiac stem cells; nuclear localization of GATA2 inhibits Yes-associated protein 1 (YAP1) signaling that drives osteogenesis on stiff matrices. Right: on stiff matrix the α2-integrin-ROCK-FAK-ERK1/2 axis is shown to increase RUNX2 activity, leading to osteoblast differentiation of MSCs; Ras pathway regulates phosphorylation levels of Smad1/5/8, ERK1/2 and AKT during osteogenic differentiation; the retinoic acid (RA) pathway enhances lamin-A transcription, but feedback by lamin-A indirectly modulates nuclear localization of RA receptor gamma (RARG), which can be inhibited by RA and promotes RUNX2 activity on stiff substrates; lamin-A also co-regulates SRF and YAP1 to drive osteogenesis; SRF signaling in turn affects stress fiber contractility; increased intracellular Ca^2+^ ion concentration on rigid matrix may contribute to cytoskeleton tension through the activation of MLCK. Broken lines, unknown or putative signaling; solid lines, as in published. Arrows indicate activation, blocked lines inhibition.

Notably, a cell has a maximal sensitivity for a particular value of stiffness, which depends on the cytoskeletal structure and the F-actin organization; a computational model has shown that the substrate stiffness sensitivity of a cell is a bell-shaped distribution over the physiological stiffness range [31]. Based on analysis of the cell tensegrity model, cells adapt to elastically tunable substrates by translating their peak sensitivity of substrate stiffness towards a value closer to the present substrate stiffness by actin filament bundling [31]. If microtubule compression forces determined by substrate stiffness are outside the range of the sensitivity of the cells (that is, the substrate is too stiff or too soft for the cells), the cells start reinforcing or dismantling their stress fibers by increasing or decreasing actin filament bundling [31]. Therefore, MSCs cultured on matrices of medium and high stiffness present a large spreading size, well-aligned stress fibers and enhanced focal adhesion assembly, all of which contribute to a high tension state, and promote myogenic and osteogenic differentiation. Conversely, diffuse actin filament networks and small spreading area associated with a relatively poorly defined actin cytoskeleton and focal adhesion assembly are found if MSCs are grown on low stiffness matrices, leading to a neuronal phenotype [6,24] (Figure 2A).

As indicated by accumulating evidence, we speculate that the cytoskeletal-based feedback loop to reach maximal sensitivity to stiffness dominates cellular mechanotransduction. According to a cell tensegrity model-based study [31], at the beginning of mechanosensing, the initial tension caused by actomyosin-modulated contractility not only allows adherent cells to exert traction stress on surrounding substrates, but also causes microtubules inside cells to experience a resisting compression force [31]. Once cells exert traction stress and adhere to the substratum, a resultant force modulated by matrix stiffness directly acts on the microtubules as another compression force [31]. Simultaneously, the initial tension caused by acto-myosin contraction and the opposing compressive forces exerted by microtubules may also be transmitted into the nucleus through the cytoskeletal network. And these forces can be resisted by the mechanosensitive intermediate filament protein lamin-A on the basis that lamin-A levels in nuclei of stem cells correlate positively with increasing ECM stiffness [32,33]. More importantly, lamin-A knockdown facilitates MSC differentiation towards low-stress, adipogenic phenotypes on soft substrates, whereas lamin-A overexpression promotes osteogenic phenotypes with high cytoskeletal stress or tension on stiff matrix [34]. In turn, lamin-A is likely to bind nuclear actin and influences cell contractility by regulating serum response factor (SRF) pathways, which is known to promote the expression of stress fiber-associated proteins [35,36]. Therefore, lamin-A could also sense the microtubule compression force and contribute to cytoskeletal organization through SRF pathways. Additionally, the microtubule compression force may be perceived and balanced by other proteins, such as vinculin and vimentin, both of which have been shown to have important roles in the mechanotransduction of mechanical stiffness [26].

Following initial focal adhesion complex formation, the resultant forces from myosin-dependent traction stress on the substratum may lead to the recruitment and activation of mechanosensitive integrin [37]. Activated integrin stimulates Rho GTPase and the downstream target protein Rho associated kinase (ROCK) to further activate MLCK. Subsequently, activated MLCK in turn mediates actin filament polymerization and actomyosin-driven contraction to generate proper cytoskeleton tension, which modulates the development and maturation of focal adhesions [37,38]. Alternatively, mechanosensitive ion channels on the cell membrane may form a molecular complex with stress fibers and focal adhesions, and convert substrate stiffness into electrical and calcium signals followed by the phosphorylation of MLCK, causing the reorganization of the actin cytoskeleton [39]. Altogether, all these matrix stiffness-induced changes in actomyosin contractility, microtubule compression force, lamin-A level, ion channels and integrin-mediated RhoA-ROCK pathways could contribute to integrin binding and cytoskeletal organization, and ultimately change cell mechnosensitivity.

## Nuclear lamin circuit

The mechanosensitive lamin-A gene circuit appears to be an important modulator of diverse transcriptional pathways, including those involving retinoic acid (RA), sterol regulatory element-binding transcription factor 1 (SREBP1), SRF, and Yes-associated protein 1 (YAP1), facilitating matrix stiffness-driven differentiation of MSCs [34]. Lamin-A can suppress MSC adipogenesis on soft substrates by decreasing the nuclear level of the adipogenic transcription factor SREBP1 [34]. Furthermore, RA inhibits osteogenesis on stiff substrate, whereas an RA antagonist not only augments osteoblast differentiation but increases lamin-A levels as well [34]. Thus, it is likely that the RA pathway, which is downstream of matrix stiffness, modulates lamin-A transcription. Meanwhile, RA signaling or nuclear localization of retinoic acid receptor gamma (RARG) may be indirectly regulated by feedback from lamin-A, as low lamin-A favors the highest cytoplasmic levels of RARG on soft substrates and *vice versa* [34]. Lamin-A also influences SRF and YAP1 (a member of the Hippo signaling pathway) to drive MSC differentiation fate. Lamin-A overexpression in MSCs on stiff matrices is accompanied by the enrichment of YAP1 at the nuclear envelope [34]. Moreover, YAP1 concentrates at the cytoplasm during MSC adipogenesis, whereas it translocates into the nucleus during osteogenesis [40].

## Crosstalk network determines stem cell fate

A complex and interconnected network of crosstalk between multiple signals triggered by matrix stiffness affects gene expression and the determination of stem cell fate (Figure 2B). MSC neurogenic differentiation on soft substrates is modulated by blocking of the BMP/Smad signaling pathway. Soft matrix promotes β1 integrin internalization, which enhances BMPR endocytosis and further leads to the expression of neuronal genes, including microtubule associated protein 2, neurofilament protein light chain and nestin [17]. Furthermore, inhibition of extracellular signal-regulated kinase (ERK)/mitogen-activated protein kinase (MAPK) signaling regulates keratinocyte differentiation of epidermal stem cells on soft substrates [41]. It has been shown that reduced clustering of integrins (β1 integrin) and decreased activation of ERK/MAPK signaling may further stimulate Jun N-terminal kinase (JNK) to increase AP-1 activity, which leads to keratinocyte differentiation [41].

The endothelial cell differentiation of cardiac stem cells is induced by matrix stiffness replicating that of the myocardium (12 to 16 kPa) in a RhoA-independent manner, as indicated by increasing expression of platelet endothelial cell adhesion molecule-1 and vascular endothelial growth factor receptor 2 [42]. Medium stiffness acts through β1 integrin, causing a reduction of p190RhoGAP, and subsequently resulting in upregulation and nuclear translocalization of the endothelial-specific transcription factors GATA2 and TFII-1 independently of RhoA activation in cardiac stem cells [42]. Moreover, GATA2 expression regulated by p190RhoGAP reduces the level of YAP as part of a nuclear lamin circuit [42]. Myogenic lineage commitment of MSCs on medium substrates (10.2 kPa) is regulated by the RhoA-ROCK-dependent cytoskeleton tension with expression of MYOD, GATA4, α-sarcomeric actin and myosin heavy chain [12,21]. However, the signaling pathway necessary for myogenesis that is triggered by the proper cellular tension needs to be clarified in future work.

MSC osteogenesis on stiff matrices is guided by the interplay between FAK and RhoA/ROCK signaling, BMP/Smad and Ras-mediated signaling. MSCs accommodate to ECM stiffness via an α2-integrin-ROCK-FAK-ERK1/2 axis that promotes RUNX2 activity, eventually leading to osteogenic fate [13]. The BMP/Smad signal pathway may also influence osteogenesis induced by stiff substrates, as suppression of the phosphorylation and nuclear translocation of Smad1/5/8 inhibits osteogenic gene expression of MSCs on BMP-2 mimetic peptide-grafted substrates with similar stiffness to muscle or osteoid [24]. Furthermore, matrix stiffness-driven MSC osteogenesis is also modulated by the Ras pathway associated with high phosphorylation levels of Smad1/5/8, ERK and AKT [43]. The Ras-mediated signaling acts as a master switch in signaling transduction since it orchestrates the activity of multiple signaling molecules, including ERK, phosphatidylinositol-3-kinase/AKT and Smad, to regulate diverse cellular functions [44]. One of the well studied pathways that activate Ras and the subsequent cellular response is through ligand binding to a receptor tyrosine kinase, which is linked to Ras via two proteins, Grb2 and SOS. Inhibition of Ras by a dominant negative mutant results in the attenuation of Smad, ERK and AKT, as well as a reduction in osteogenic marker expression by MSCs [43]. However, the Ras pathways are essential but not sufficient for matrix stiffness-regulated osteogenesis, as constitutively active Ras has little effect on Smad and AKT phosphorylation or the osteogenic markers [43]. Therefore, other signaling networks involved in these processes need to be studied in the future.

## A rapid shift from original mechanosensing to *de novo* cell-derived matrix sensing in three-dimensional cultures

**Figure 3:**
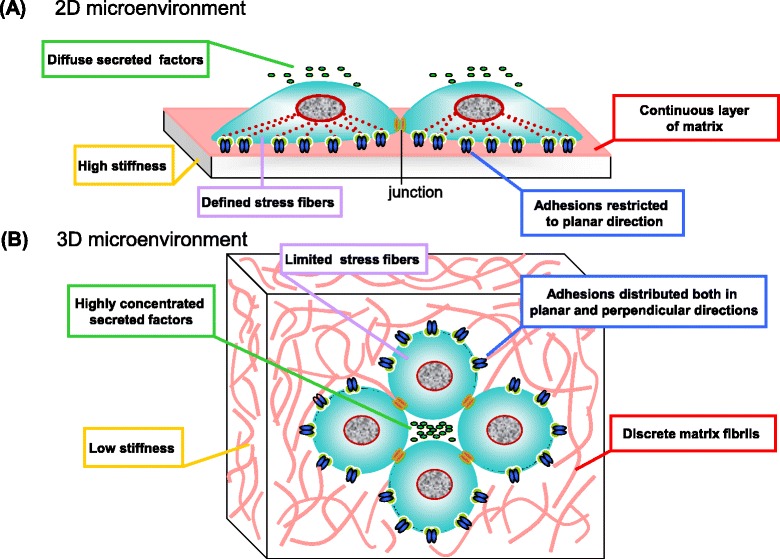
Discrepancy between two-dimensional and three-dimensional microenvironments. **(A)** Cells on a rigid two-dimensional (2D) surface coated with a continuous layer of matrix develop focal adhesions and stress fibers at the basal surface. Cell-matrix interactions are restricted to the planar direction and gradients of secreted factors are absent. **(B)** Inside a three-dimensional (3D) environment, cells display poorly defined stress fibers due to the softness of biomaterials. Cell adhesions are distributed both in the planar and perpendicular directions with discrete matrix fibrils, and secreted factors can be highly concentrated.

Most current investigations are derived from *in vitro* two-dimensional cell culture models that might not truly reflect the characteristics of the cells in physiological three-dimensional environments as two-dimensional cell-matrix interactions are bidirectional, while three-dimensional ones are omnidirectional. Thus, it might be more suitable to study the mechanism of matrix stiffness mechanotransduction in three-dimensional studies. In fact, inside a three-dimensional microenvironment, the softness and curvature of biomaterials hinder the formation of actin stress fibers and cell spreading, and secreted factors can be highly concentrated relative to two-dimensional conditions; thus, it is not surprising that stem cells may sense ECM stiffness in a different way [45] (Figure 3). More and more researchers have shown that the effects of matrix stiffness on stem cell morphology and differentiation are different in two-dimensional versus three-dimensional cultures (Table 2).

**Table 2:** **Effects of matrix stiffness on stem cells in two dimensions versus three dimensions**

	**Stem cell types**	**Morphology and cytoskeletal organization**	**Differentiation**
Two-dimensional	ESCs	Col I-coated PDMS: 41 to 260 kPa, lower cell perimeter and less spreading; 2,700 kPa, higher cell perimeter, increased spreading and more stress fibers [62]	HyA gel: 1.2 kPa, pancreatic beta cell [9].
			Col I-coated PDMS: 2,700 kPa, mesendoderm cell and osteoblast [62]
	MSCs	FN-coated PAAm gel: 1 to 2 kPa, rounded and unspread; ≥5 kPa, well spread and amassed a large number of stress fibers [63]	Col I gel and Col I-coated gel, 1 kPa, adipocyte and chondrocyte; 15 kPa, smooth muscle cell [64]
		Col I gel and Col I-coated PAAm gel: 1 kPa, less spreading and fewer stress fibers; 15 kPa, more spreading and extensive stress fibers [64]	Col I-coated PAAm gel: 0.1 to 1 kPa, neuron; 8 to 17 kPa, myoblast; 25 to 40 kPa, osteoblast [6]
		Col I-coated PAAm gel: 0.1 to 1 kPa, branched and filopodia-rich; 8 to 17 kPa, spindle-shaped; 25 to 40 kPa, polygonal [6]	Sulfonate-coated PAAm:1 kPa, chondrocyte [25]
		Sulfonate-coated PAAm; 1 kPa, round shapes with less stress fibers but more cortical actins; 150 kPa, spread out with strong expression of stress fibers [25]	
	ASCs	Adipose matrix-coated PAAm gel: 2 kPa, compact, rounded and smaller aspect ratio; 20 to ~40 kPa, more spreading and larger aspect ratio [61].	Adipose matrix-coated PAAm gel: 2 kPa, adipocyte [61]
	Cardiac stem cells	FN-coated PAAm gel: 12 to 16 kPa, more rounded and forming organized cellular networks with rounded gaps; FN-coated glass: fibroblast-like [42]	FN-coated PAAm gel: 12 to 16 kPa, endothelial cell [42]
	Mammary progenitor cells	Col I-coated PAAm gel: 0.1 kPa, cobblestone; 4 kPa, more elongated [60]	Col I-coated PAAm gel: 0.1 kPa, luminal epithelial cell; 4 kPa, myoepithelial cell [60]
Three-dimensional	ESCs	Col I gel and matrigel: 0.02 to 0.3 kPa, less neurite outgrowth; 0.5 to 1 kPa, branching and more neurite outgrowth [65]	PLLA, PLGA, PCL coated matrigel: 50 to 100 kPa, ectoderm cell; 100 to 1,000 kPa, endoderm cell; 1,500 to 6,000 kPa, mesoderm cell [7]
			Col I gel and matrigel: 0.02 to 0.3 kPa, glial cell; 0.5 to 1 kPa, neuron [65]
	MSCs	Col I-HyA gel: 1 kPa, spherical and formed colonies; 10 kPa, flattened [66]	PEG-silica thixotropic gel: 7 Pa (τy)^a^, neuron; 25 Pa (τy) , myoblast; 75 Pa (τy), osteoblast [54]
		PEG gel: 0.2 to 59 kPa, spherical, lack of well-defined actin filaments and microtubules [46]	HyA hydrogel: 3.5 kPa, chondrocyte; 53.6 kPa, hypertrophy [48]
		Gtn-HPA gel: 0.6 to 2.5 kPa, less spreading, less organized cytoskeletons; 8 to 12 kPa, greater spreading, more organized cytoskeletons and more stable focal adhesions [67]	Col I-HyA gel: 1 kPa, neuron; 10 kPa, glial cell [66]
			PEG gel: 0.2 to 59 kPa, osteoblast [46]
			Gtn-HPA gel: 0.6 to 2.5 kPa, neuron; 8 to 12 kPa, myoblast [67]
		Alginate gel containing RGD: 5, 10, 22, 45, 110 kPa, grossly spherical and morphology are not strongly correlated to matrix stiffness [47]	Col I-coated PEG nanofiber: 2 to 5 kPa, endothelial cell; 8 to 15 kPa, smooth muscle cell [68]
		Col I-coated PEG nanofiber: 2 to 5 kPa, less polarized; 8 to 15 kPa, striated, elongated and greater spreading [68]	PCL nanofiber: 7,100 kPa, chondrocyte; PES-PCL nanofiber: 30,600 kPa, osteoblast [59]
		PCL nanofiber: 7,100 kPa, rounded; PES-PCL nanofiber: 30,600 kPa, spread and higher stress fiber density [59]	
	Myoblasts	TG-Gtn gel: 2 kPa, elongated and branched, mesh-like or extended actin filaments; 14 to 32 kPa, dot-like actin microfilaments with filopodia [49]	TG-Gtn gel: 14 to 32 kPa, osteoblast [49]

^a^τy (liquefaction stress): the minimum shear stress required to liquefy the gel is used to measure the substrate stiffness. ASC, adipose-derived stem cell; Col I, collagen I; ESC, embryonic stem cell; FN, fibronectin; Gtn-HPA, gelatin-hydroxyphenylpropionic acid; HyA, hyaluronic acid; MSC, mesenchymal stem cell; PAAm, polyacrylamide; PCL, poly(ε-caprolactone); PDMS, polydimethylsiloxane; PEG, poly(ethylene glycol); PES, poly(ether sulfone); PLGA, poly(lactic co-glycolic acid); PLLA, poly(L-lactic acid); RGD, arginine-glycine-aspartate; TG-Gtn, transglutaminase-gelatin.

Myosin-based cytoskeletal tension is unlikely to be involved in three-dimensional mechanosensing, but integrin binding still plays an important role according to studies using pharmacological cytoskeletal inhibitors in three-dimensional culture (Table 1). In three-dimensional scaffolds with stiffness ranging from low (0.2 kPa) to high (59 kPa), MSCs undergoing osteogenic differentiation lack pronounced actin filaments and microtubules [46], in contrast to previous reports in two-dimensional culture showing more well-established actin stress fibers with larger cell spreading size, along with osteogenesis on stiffer versus soft substrates [6]. When encapsulated in three-dimensional scaffolds, MSCs initially express no adhesive peptides or ECM proteins, but they produce their own matrix over time, as evidenced by a great increase in fibronectin found around cells. Moreover, blocking of integrin-ECM interaction by RGDS inhibits MSC osteogenesis in three-dimensional scaffolds [46]. Thus, integrin binding to the *de novo* ECM is essential for stem cell mechanosensing in three-dimensional cultures, in accordance with previous work [47]. Nevertheless, myosin-based cytoskeletal tension and microtubules may not play a part in matrix stiffness-induced MSC differentiation in three-dimensional conditions, as disruption of mechanosensing elements, including actin filament, microtubule formation, non-muscle myosin II, MLCK and ROCK, does not largely change their expression of osteogenic markers [46]. Similarly, three-dimensional hydrogels with lower stiffness (approximately 3.5 kPa) promote MSC chondrogenesis, whereas stiffer hydrogels (approximately 53.6 kPa) lead to hypertrophic differentiation with more matrix mineralization, which resembles the terminal differentiation of growth-plate hypertrophic chondrocytes [48]. The greater hypertrophic differentiation may not be modulated by a force-sensing mechanotransduction mechanism because blocking of ROCK and myosin II has no obvious effect on major hypertrophic marker expression in stiff hydrogels [48]. In another study on myoblasts, cells underwent osteogenesis in medium and stiff substrates, accompanied by spherical and dot-like actin microfilaments with more micron-sized cortical protrusions that are found to enter the surrounding matrices via β1 integrin [49]. These results again suggest that integrin binding is involved in three-dimensional mechanosensing processes.

Importantly, newly self-synthesized cell-derived ECM probably acts as a key regulator in determining stem cell fate. Rapid osteogenesis of myoblasts occurs on stiff (32 kPa) substrates comparable to native bone tissue, as shown by highest calcium deposition and late osteogenic marker expression [49]. Interestingly, on medium substrates most cells express only early osteogenic transcripts, but they also differentiate into osteoblast-like cells at a later point in time. This phenomenon may occur because they assemble and deposit their own matrix matching the bone microenvironment [49]. Furthermore, soft hydrogels contain higher amounts of proteoglycans and collagen II, both of which are known to facilitate chondrogenesis but suppress hypertrophic differentiation and matrix calcification [50]. Additionally, more overall accumulation and uniform spatial distribution of glycosaminoglycan in soft scaffolds can bind and concentrate some hypertrophy inhibitory growth factors such as transforming growth factor βs [51]. These may be the reason why MSCs favor chondrogenic differentiation over hypertrophic differentiation in three-dimensional soft environments [48]. Extremely soft (<0.12 kPa) three-dimensional hydrogel preferentially induces cell aggregation and osteogenic differentiation of MSCs, even without arginine-glycine-aspartate (RGD) ligands [52]. In soft RGD-alginate matrices MSCs produce their own fibronectin around cell aggregates, associated with high expression of the α5 integrin subunit, and they are likely to effectively reinforce the hydrogel stiffness by increasing local matrix density via network contraction and fibronectin assembly [53]. Consequently, depending on cellular rearrangement, a new mechanical and biochemical microenvironment is created by local matrix stiffening, cell aggregation and α5β1 integrin-mediated fibronectin binding. In turn, all of these changes may control MSC commitment, as stiffer matrix and cell aggregation have a positive correlation with osteogenic differentiation [54], and α5 integrin signaling is known to monitor stem cell osteogenesis [55,56].

Altogether, interactions of stem cells entrapped in a three-dimensional environment with the initial matrix become less dominant over time, but newly self-synthesized ECM where cells reside turns into a more crucial mediator of stem cell fate. Although a mechanotransduction mechanism dependent on myosin-based cytoskeletal tension and microtubules seems not to work in three-dimensional conditions, the early interaction with the intrinsic stiffness of the original matrix through integrin binding is clearly decisive. A putative shift from initial mechanosensing to *de novo* cell-derived matrix sensing in three-dimensional conditions may be the mechanism by which stem cells dynamically perceive their microenvironment and make fate decisions.

## Conclusion and future perspectives

It is becoming increasingly clear that substrate stiffness plays a central role in determining stem cell fate in two- and three-dimensional cultures (Table 2), while the mechanisms of mechanotransduction remain unclear. Based on most results acquired from *in vitro* two-dimensional cell culture models, cytoskeleton-based feedback loops make cells reach maximal mechanosensitivity towards matrix stiffness, and the lamin-A gene circuit plays a vital role in modulating diverse transcriptional pathways. Multiple signaling crosstalk triggered by matrix stiffness finally changes gene expression and determines stem cell fate. However, cellular mechanosensing is intricate and complex in that substrate stiffness is only one of multiple mechanical stimuli that stem cells may experience *in vivo*. Matrix stiffness may work in concert with other mechanical cues to regulate and coordinate stem cell behaviors, such as hydrostatic pressure and fluid shear stress [26,57]. Nevertheless, as *in vivo* most cells reside within a complex environment containing multiple ECM components, a medley of cell populations and mixtures of cell-secreted factors, three-dimensional cell culture models recapitulate the natural tissue environment more closely. It is highly likely that a very different regulatory mechanism of stem cell differentiation by substrate stiffness exists in three-dimensional culture. We speculate stem cells dynamically perceive their microenvironment through a shift from initial mechanosensing to newly self-synthesized matrix sensing in a three-dimensional extracellular milieu, but the specific mechanism needs further research.

Although the value of three-dimensional cell culture has been proved repeatedly, research to identify stem cell responses to substrate stiffness in three-dimensional conditions still faces many challenges. One of the major challenges is the lack of suitable substrates with physiologically relevant properties. For instance, although thixotropic PEG-silica gels provide an inert environment for stem cell culture, which avoids the complication of biological signaling from a biologically derived matrix, their application as scaffolds are limited due to their insufficient stiffness [54]. On the contrary, silk fibroin has an inherent tendency to form stiff substrates (>1,000 kPa), which limit its elastomeric biomaterial applications [58]. Meanwhile, transglutaminase cross-linked gelatin may be a promising biomaterial due to its mechanical stiffness (2.5 to 100 kPa), suitable for supporting cell differentiation during tissue regeneration [49]. Furthermore, nutrients and gas, which also are important variables that influence stem cell fates in three-dimensional conditions, diffuse freely through these gels, regardless of the gel porosity or stiffness. However, although it is possible to modify the substrate stiffness by varying the degree of hydrogel cross-linking or its composition, it is impossible to eliminate the discrepancies in surface chemistry, topography and porosity. Recently, electrospinning has been utilized to create mechanically distinct scaffolds that have identical surface chemistries and microstructures, allowing the dissection of the effect of matrix stiffness on stem cell fate decisions in three dimensions [59].

Furthermore, cell density or cell-cell interactions might also influence the findings in three dimensions. To minimize the effects of cell density, some researchers culture cells in scaffolds for a short period until the cell density reaches approximately 70 to 80% confluence [59]. During the cellular processes the mechanical properties such as stiffness, topography and porosity may dynamically change and be distributed unevenly across the gels because of the possible degradation of gels and the endogenous ECM assembly [47]. Therefore, it is urgent to further assess the dynamic changes in mechanical properties of the substrates, and biomaterials with mechanical properties that vary across a gradient may create an ideal platform.

## Abbreviations

BMP: Bone morphogenetic protein
BMPR: Bone morphogenetic protein receptor
ECM: Extracellular matrix
ERK: Extracellular signal-regulated kinase
FAK: Focal adhesion kinase
GAP: GTPase activating protein
JNK: Jun N-terminal kinase
MAPK: Mitogen-activated protein kinase
MLCK: Myosin light chain kinase
MSC: Mesenchymal stem cell
RA: Retinoic acid
RARG: Retinoic acid receptor gamma
RGD: Arginine-glycine-aspartate
RGDS: Arginine-glycine-aspartate-serine
ROCK: Rho associated kinase
SREBP1: Sterol regulatory element-binding transcription factor 1
SRF: Serum response factor
YAP1: Yes-associated protein 1
